# Hepatocyte nuclear factor 1 beta: A perspective in cancer

**DOI:** 10.1002/cam4.3676

**Published:** 2021-02-13

**Authors:** Shubhra Chandra, Srilakshmi Srinivasan, Jyotsna Batra

**Affiliations:** ^1^ Institute of Health and Biomedical Innovation and School of Biomedical Sciences Australian Prostate Cancer Research Centre‐Queensland Queensland University of Technology Brisbane QLD Australia; ^2^ Translational Research Institute Woolloongabba QLD Australia

**Keywords:** cancer, hepatocyte nuclear factor 1beta, splice variants, transcription factor

## Abstract

Hepatocyte nuclear factor 1 beta (HNF1 β/B) exists as a homeobox transcription factor having a vital role in the embryonic development of organs mainly liver, kidney and pancreas. Initially described as a gene causing maturity‐onset diabetes of the young (MODY), HNF1β expression deregulation and single nucleotide polymorphisms in HNF1β have now been associated with several tumours including endometrial, prostate, ovarian, hepatocellular, renal and colorectal cancers. Its function has been studied either as homodimer or heterodimer with *HNF1α*. In this review, the role of *HNF1B* in different cancers will be discussed along with the role of its splice variants, and its emerging role as a potential biomarker in cancer.

## Graphical abstract

In this review, the role of HNF1B in different cancers has been discussed along with the role of its splice variants, associated functions and the emerging role of its epigenetic regulation.

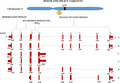

## 1 INTRODUCTION

Hepatocyte nuclear factors (HNFs) exist as an assembly of transcription factors that have a vital role in the expression regulation of genes pertaining to the liver.^1^ Conversely, these transcription factors are not restricted to hepatocytes, their expression has been observed in other tissues.^2^ There are four major families of HNFs namely HNF1, HNF3, HNF4 and HNF6. The HNF1 family members; HNF1‐α/A and HNF‐1β/B comprise a POU‐homeodomain and bind to DNA as homodimers or heterodimers.^3^

**FIGURE 1:**
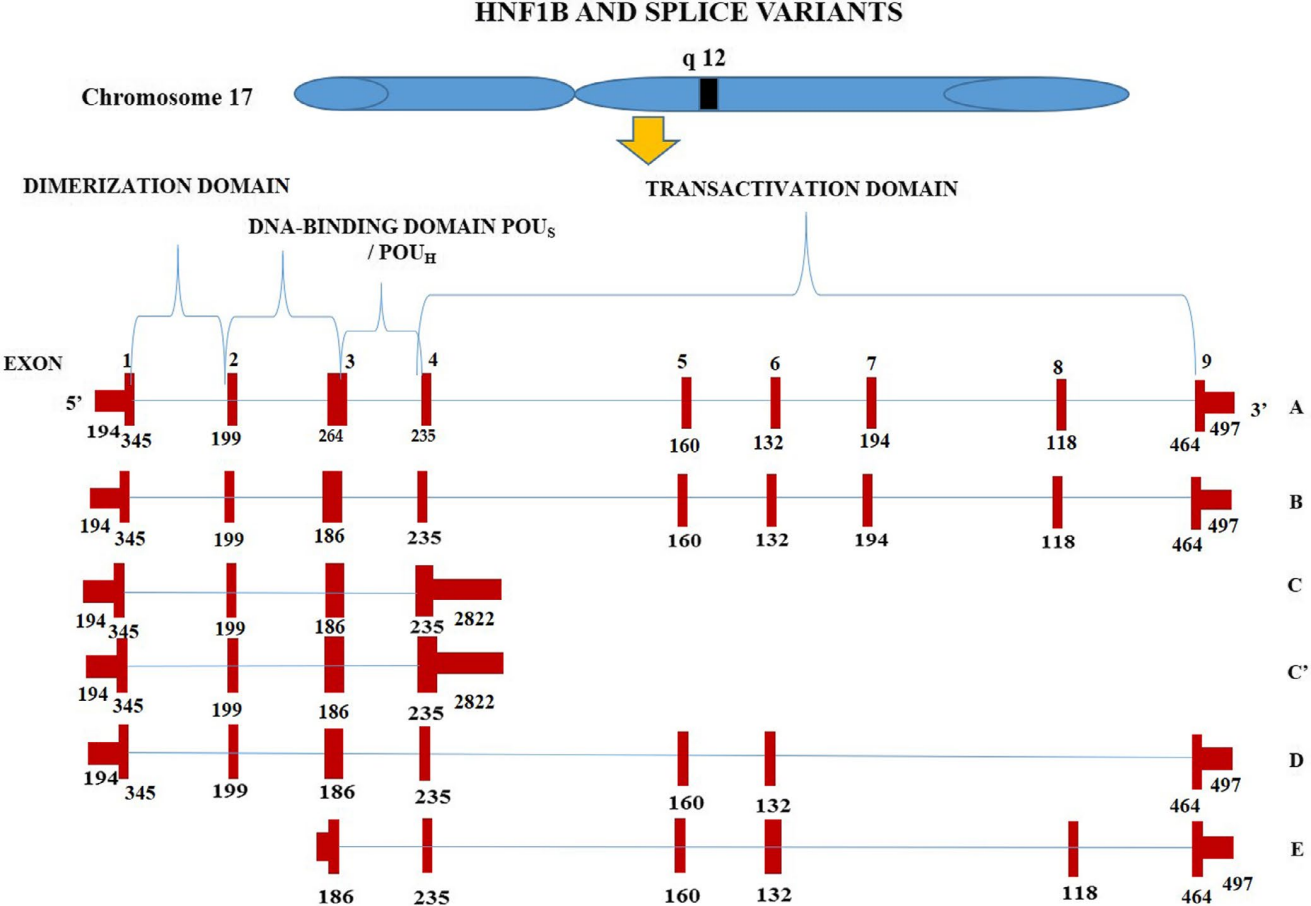
HNF1B is a transcription factor with dimerization domain, DNA‐binding domain POU_s_/POU_H_ and has six transcript variants (3 classical [A, B and D] and 3 annotated [C, C’ and E]). The exons along with untranslated regions (5’ and 3’) are demarcated with red bars along with their size in bp, respectively. *HNF1B A* and *B* transcript variants are similar with respect to all exons except for a difference of 78 bp in the third exon. Transcript variant C, lacks 7th and the 8th exon coding for transactivation domain

Hepatocyte nuclear factor 1B (*HNF1B*, *TCF2*) located on chromosome 17q12,^4^ belongs to the family of Pit‐1, Oct‐1/2, UNC‐86 (POU) homeodomain‐comprising transcription factors (Figure 1). The POU domain is bipartite (POU_H_ and POU_S_) and is comprised of two subunits separated by a short 15–55 amino acid (aa) non‐conserved region. The protein contains three predominant functional domains for dimerization, DNA binding and transactivation, respectively. HNF1B protein is anticipated to have an alpha‐helical structure which could lead to the formation of a coiled structure.^5^ Wu et al studied different domains of HNF1B in the process of nephrogenesis and identified dimerization domain, homeodomain and a conserved 26 amino acid segment between the POU_S_ and POU_H_ domain found in B variant of *HNF1B* to interfere with pronephric development.^6^ The 26 aa fragment may interact with other transcription factors or may alter the protein structure of HNF1B. This emphasises the role of different domains in *HNF1B* splice variants and their role in determining the function of the gene.

In humans, mutations in *HNF1B* have been recognised with multisystem phenotype. *HNF1B* mutations were first defined as an intermittent genetic basis of maturity‐onset diabetes of the young (MODY).^7^ It includes a set of ailments that are normally identified by autosomal dominant inheritance, early onset of diabetes mellitus, commonly before 25 years of age, and pancreatic beta‐cell disruption. Renal cysts were found to be the main clinical aspect with *HNF1B* mutations and the linkage of renal cysts with diabetes steered to the description of renal cysts and diabetes syndrome. Supplementary phenotypes detected in patients include urogenital tract anomalies, condensed exocrine function, anomalous liver function tests, pancreatic hypoplasia, biliary disorders, hypomagnesaemia, hyperuricaemia and early‐onset gout.^8^, ^9^

Ever since the advent of single nucleotide polymorphism (SNP) genotyping arrays, genome‐wide association studies (GWAS) are performed to detect numerous loci associated with multiple diseases, including cancer. The vast majority of these SNPs are in intergenic or intronic regions influencing gene regulation..^10^, ^11^, ^12^ Fine‐mapping analysis has revealed an complex genetic architecture at *HNF1B* gene loci, relating to different cancer types.^13^ The current review summarises the emerging role of *HNF1B* and its association with cancer risk in several tumours, and its importance in tumorigenesis.

## 2 GENETICS AND EPIGENETICS OF *HNF1B*

Genetic predisposition in human cancer has always been an intriguing area of study, wherein population‐based studies have led to genetic models being implemented to explain patterns of the rate of disease occurrences in specific population.^14^ GWAS have identified SNPs in *HNF1B* associated with endometrial cancer (EC) risk in European ancestry and identified rs4430796 SNP as an EC susceptible locus near *HNF1B* ^15^ and was replicated in multiethnic replication studies.^16^, ^17^ Nevertheless, these studies emphasized the requirement of large cohorts and stringent tumour stage grouping to classify novel genetic polymorphisms linked with EC vulnerability. Setiawan et al. in a large case‐control cohort study on *HNF1B* variants identified rs7501939 SNP in addition to the rs4430796 SNP and confirmed their association with EC risk, demonstrating the association of this locus with both EC type I as well as II tumours.^18^ Fine‐mapping studies and laboratory analysis of the *HNF1B* locus, identified potentially causal variants that may mediate the risk of EC. The SNPs detected in this study were also reported to be associated with *HNF1B* methylation.^13^ The minor allele of rs11263763 and rs8064454 SNPs are correlated with decreased *HNF1B* promotor activity suggesting the risk observed at the *HNF1B* locus is likely mediated due to altered *HNF1B* gene expression.^13^

The initial report on *HNF1B* association with prostate cancer risk came from a GWAS in Iceland^4^ and was later replicated in the USA and UK populations^19^, ^20^ which showed two distinct prostate cancer risk‐associated loci on chromosome 17q. SNP rs4430796, in *HNF1B*, was strongly linked to prostate cancer risk and was one of the first loci to be identified for prostate cancer.^21^ Another independent SNP, rs11649743, found on chromosome 17q12 was later established to correspond with prostate cancer risk.^21^ Widespread fine‐mapping studies have been conducted in the region 17q12 and have confirmed the previously documented signals associated with prostate cancer risk. A fine‐mapping study in European ancestry indicated the role of five SNPs (rs4430796, rs4794758, rs3094509, rs7405696 and rs1016990) as the best predictors for prostate cancer risk in *HNF1B* gene loci.^22^ Another study conducted by Olama et al. identified two novel causal variants, rs11263763 and rs718961 in the *HNF1B* region, along with rs2229295 within 3’UTR to be another potential casual variant for further investigation.^23^ The novel SNPs, rs11263763 in the first intron and rs718961 in the fourth intron overlapping with several bio features were regarded as promising candidates with functional impact. Further, Zhang et al. discovered the loci linked with prostate cancer in North Chinese inhabitants pointing out that the risk allele of the rs4430796 SNP can be associated with high PSA levels.^24^ Another study on non‐Hispanic white families by Levin et al. showed SNPs rs4430796 and rs7501939 to be potential risk alleles in primary prostate tumours.^25^ Machiela et al when examining common variants between type‐2 diabetes (T2D) and prostate cancer found rs757210 in *HNF1B* to be significantly associated with prostate cancer risk. The data presented by the authors found a shared genetic association between T2D and prostate cancer risk.^26^ Additionally, diabetogenic variants were also shown to be associated with multiple myeloma. A study by Rios‐Tamayo et al. investigated the impact of these diabetogenic variants on the overall survival of multiple myeloma patients.^27^ The intronic rs7501939 SNP was reported to be significantly associated with poor survival in multiple myeloma patients. The authors argued that the functional effects of this SNP could be mediated by non‐insulin‐dependent mechanisms contributing to tumour progression. Although there was no supporting evidence for this hypothesis, in‐depth investigation is required into associating these risk variants with differential regulation of transcript variants/isoforms which may have tumour suppressor and/ or oncogenic roles.

SNPs at *HNF1B* gene loci are also associated with biochemical failure and tumour aggressiveness. Logistic regression analysis in Korean patients (240 prostate cancer subjects and 223 controls) to evaluate the effects of 47 SNPs in *HNF1B* on prostate risk.^28^ The rs11868513 SNP was found to be more prevalent in patients with higher aggressive tumour stage compared to the low aggressive tumour stage. Strong prostate cancer risk association was observed in patients with rs4430796 and rs2074429 haplotypes with a Gleason score of ≥7. Although this study was the first in associating *HNF1B* polymorphisms with prostate cancer risk in Korean men, the statistical significance of the SNPs was limited due to the small sample size. Additionally, the comparisons were between prostate cancer patients and non‐malignant BPH patients which could have influenced the final results. Using a multilevel molecular epidemiology approach in patients with prostatectomy and biochemical failure,^29^ the rs4430796 SNP was found to be significantly associated with increased biochemical failure (*p* = 0.02), highlighting the biological relevance of this SNP in disease progression. All these studies highlighting the use of SNPs in identifying biochemical failure or tumour aggressiveness, seem to be limited in their approach due to their analysis in small cohorts. Validation in large cohorts may provide a strong basis for using these data in clinical applications.

*HNF1B* has also been found to be associated with testicular germ cell tumour. Researchers in Sweden performed a GWAS by genotyping 610,240 SNPs from patients and control samples from Norway and Sweden.^30^ After finding no novel associations in the discovery phase, a replication study found 15 novel regions to be associated with the disease. Genome‐wide significance was obtained for the SNP at *HNF1B* gene loci. Although some of the known major risk loci (*KITLG*, *SPRY4*, *BAK1* *and* *DMRT1*) were also implicated with this cancer risk, some of the low penetrant risk alleles such as rs7501938 SNP also need to considered for a better understanding of the genetic cause of this disease.

Despite the great knowledge of mutations in *HNF1B* in MODY, few studies have highlighted the role of mutations in cancer. Nemejcova et al while understanding the genetic changes of *HNF1B* in endometrial lesions, revealed truncated variants in ovarian clear cell carcinoma (CCC).^31^ The study identified 4 sequence variations and one missense mutation in ovarian CCC patients. *In silico* analysis suggested a nonsense mutation (p.Gln152X) to lead to premature translation termination and another missense variant in the DNA binding domain (p.Ala283Val) to have a damaging effect on the function of the protein (Refer to Table 1).

**TABLE 1:** *HNF1B* variants association with different cancers

Cancer	GWAS SNP	Location	Reference
Endometrial	rs4430796,	Intron_variant	13, 16, 18
	rs7501939,		
	rs11263763,		
	rs8064454,		
Prostate	rs4430796,	Intron_variant	4, 28
	rs11649743,		
	rs4794758,		
	rs3094509,		
	rs7405696,		
	rs1016990,		
	rs11263763,		
	rs2229295,		
	rs718961,		
	rs757210,		
	rs11868513,		
	rs2074429,		
Testicular germ cell	rs7501938	Intron_variant	^30^
Ovarian CCC	rs757210	Intron_variant	^32^

### 2.1 HNF1B and epigenetics

Genetic modifications and Epigenetic mechanisms influence carcinogenesis. Identification of mutations and its associated effect on the epigenome through next‐generation sequencing has broadened our understanding of cancer regulatory pathways affecting cellular characteristics.^33^ DNA methylation is associated with gene transcription regulation.^34^ In a study conducted to investigate the role of *GATA4* and *HNF1B* methylation in Ovarian cancer (OC) patients, around 32.8% *HNF1B* methylation positive patterns were observed in OC tissue samples compared to controls.^35^ Unmethylated *HNF1B* regions in control samples correlated with higher survival rate in patients suffering from OC. Overall, the methylation pattern for both genes was found to be 65.6%, highlighting the potential of these genes as methylation prognostic panel markers.^35^

The methylation profile of *HNF1B* was further characterised by Terasawa et al in OC cell lines and tissue samples.^36^ Epigenetic silencing of the *HNF1B* gene was observed in the cell lines due to methylation, decreasing the expression of *HNF4α* suggesting the role of hepatocyte nuclear factor network of genes in tumours. The study also reported the role of histone deacetylation organized with methylation to be involved in tumorigenesis in ovarian cancer. Additionally, this group also highlighted *HNF1B* methylation patterns in colorectal, gastric and pancreatic cell lines, all of which showed silencing of *HNF1B* gene expression due to methylation. Moreover, in breast tumours, methylation of homeobox genes including *HOXB13* and *HNF1B*, CpG islands has been found to be significant and more pronounced compared to normal samples.^37^ Early‐stage breast cancer samples exhibited 73% methylation patterns establishing the role of *HNF1B* methylation with epigenetic reprogramming and carcinogenesis in breast tumours, confirming the role of the epigenome in carcinogenesis. These results give an indication of the epigenetic mechanisms involved with homeobox genes including *HNF1B* in tumorigenesis.

Distinct DNA methylation patterns were observed for the different histological tumour subtypes, underlying divergent mechanisms involved in ovarian cancer subtypes. Unmethylated *HNF1B* promoter displayed expression of the protein in clear cell tumours whereas serous tumours which displayed methylation at *HNF1B* lacked the protein expression.^38^ The research also suggested a negative correlation between *HNF1B*‐promoter methylation and CpG island hypermethylation. After identifying causal SNPs in the *HNF1B* region, the SNP‐*HNF1B* promoter DNA methylation was found to overlap with a polycomb repressive complex 2 mark in serous ovarian cancer. Molecular signatures like these (HNF1B status or CIMP) may help classify subtypes of clear cell carcinomas. A study to understand DNA methylation in colorectal cancer identified *HNF1B* methylation in colorectal patients. *HNF1B* was found to be highly methylated in addition to 4 other genes *(RUNX3*, *PCDH10*, *SFRP5*, *IGF2)*, which repressed its expression^39^ indicating varying extent of methylation for *HNF1B* gene. In clear cell carcinoma, a pattern of hypomethylation has a positive correlation to increased expression suggesting an oncogenic role in this disease. Alternatively, the negative correlation in methylation: expression patterns indicate a tumour suppressor role of HNF1B in high grade serous ovarian cancer. Altogether, the methylation patterns in *HNF1B* may be used as prognostic predictors, highlighting the value of performing a pan‐cancer methylation study in a large cohort of patients.

## 3 *HNF1B* AND ITS SPLICE VARIANTS

Alternative splicing has been vastly studied in cancer, changing the landscape and functions of many oncogenes and tumour suppressor genes.^40^ It's strenuous to interpret the role for these transcripts, since the translated alternative splice exons code for critical functional domains of the encoded protein,^42^, ^43^, ^44^, ^45^ for instance, BCL2 family, wherein intron 2 of the Bcl2L1 gene, coding for Bcl‐X protein undergoes alternative splicing, the mRNA produced could have a different function such as, the classical large protein having anti‐apoptic function and the novel truncated protein lacking the BH domain has a pro‐apoptic function.^46^ Several tumour suppressor genes also undergo alternative splicing, causing, complete or partial loss of function. For example, TP53, coding for p53 protein gives rise to multiple isoforms having different protein functions.^47^ Alternative splice variants of P53 can induce cell cycle arrest in G1/G2 cell cycle phase or could activate/regulate cell cycle arrest in G2/M phases. These are few examples that highlight the scope of alternative splicing generating multiple proteins from a single gene which would result in contrasting functions impacting cell function.

Alternative mRNA splicing events are conserved amongst species. However, a substantial amount of genes identified to be alternatively spliced in human, do not form numerous transcript variants in rodents.^48^ Alternate splicing may give rise to different *HNF1A*, *B* and *HNF4A* genes and diverse isoforms (eight isoforms in *HNF1A* and four isoforms in *HNF1B*; 9 in *HNF4A*) in humans by an arrangement of differential polyadenylation sites, alternate promoter usage and alternative splicing.^49^, ^50^, ^51^ *HNF1A*, *HNF1B* and *HNF4A* transcription factors are present in a synchronized feedback circuit in majority tissues, although the specific essence of collective regulation might vary amongst tissues.^52^

**FIGURE 2:**
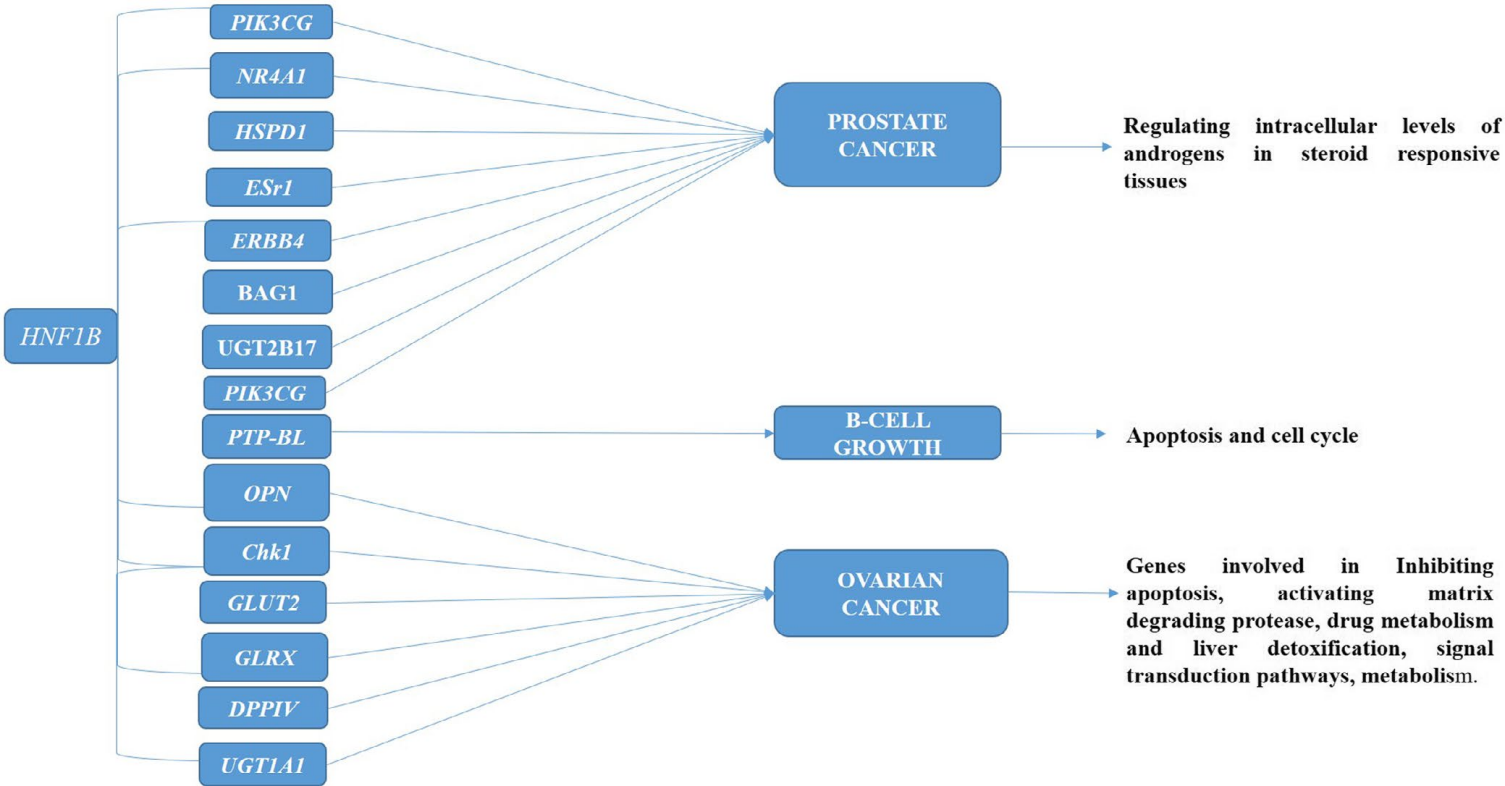
Target genes of HNF1B in various cancers/pathways

The alterations among transcript variants lead to the formation of proteins with different properties. *HNF1A* and *B* function just as dimers, so even minimal amounts of the isoforms can alter overall action *in* *vivo*.^53^ Human *HNF1B* gene encodes three transcript variants (Figure 1), *HNF1B* *(A)*, *HNF1B* *(B)* and *HNF1B* *(C)* (Figure 2). *HNF1B* (A) and *HNF1B* (B) variants are analogous structurally and hence could exhibit functional anomaly. It was observed that higher levels of *HNF1B* (C), a repressor molecule could lead to a decline in HNF1B activity in rodents. Data have suggested that *HNF1A* *(*B*)*, *HNF1A(*C*)*, *HNF4A3* *and* *HNF4A9* might have a function in human beta cells as their existence can alter MODY phenotype.^49^, ^50^ A study by Harries et al indicated *HNF1B* *(*C*)* isoform as predominant in Benign Prostate Hyperplasia (BPH), amounting to 90% of the total gene's expression. However, in prostate cancer tissues *HNF1B* *(*B*)* isoform contributed to 95% of the total HNF1B expression. *HNF1B* *(*C*)* accounted for only 3% while *HNF1B* *(*A*)* proportion did not change.^50^ *HNF1B* *(*C*)* variant has also been detected to negatively control GSTA (Glutathione‐S‐transferase A) promoter.^54^ The role of *HNF1B* splice variants needs to be well defined in cancer and a better understanding of the mechanisms might lead to a breakthrough in therapeutic applications.

## 4 HNF1B INTERACTIONS AND TARGET GENES

To understand the cellular processes, it is essential to identify the target genes affected by the transcription factor. Several studies have identified the individual target genes of HNF1B in hepatic‐cell, ovarian and kidney cell lines (Figure 2). *HNF1B* suppression has been found to downregulate the expression of the HNF family members‐including *HNF1A*, *HNF4A*, *HNF6*, *HNF3* *(HNF3α*, *β* and *γ*) in mouse liver cells. Other genes like HNF1, insulin‐like growth factor binding protein 1, Albumin, α‐fetoprotein were downregulated in mouse hepatoma cells on suppression of *HNF1B* by RNAi, whereas apolipoprotein, α1‐antitrypsin, alcohol dehydrogenase 2 and α‐fibrinogen showed increased expression.^55^ Hu et al. reported *HNF1B* as a prostate cancer risk gene with its mechanism of action assessed in a wide array of prostate cancer cell lines. Twelve genes were found to be associated with *HNF1B*, six of them (*BAG1*, *ERBB4*, *ESr1*, *HSPD1*, *NR4A1* and *PIK3CG)* mapped to KEGG pathways (Figure 2).^56^ A study understanding the regulation of the UDP glucuronosyltransferase 2B17 gene promoter found elevated expression of this gene by *HNF1B* in LNCaP prostate cancer cell line.^57^ Wang et al further demonstrated the enhancer of the zeste homolog 2 (EZH2) as one of the downstream targets of HNF1B along with its overexpression shown to be associated with prostate cancer malignancy. EZH2 binds to the *HNF1B* locus which then suppresses *HNF1B* expression in prostate cell lines, although when the function of HNF1B restored the expression of EZH2 is repressed with increased invasion and migration.^58^ Together these studies highlight the transcriptional role of *HNF1B* and its network in prostate cancer, although extensive studies on its mechanism are required to interpret its role in disease.

Overexpression of *HNF1B* in pancreatic B cells has been linked to apoptosis and cell cycle, underlying its importance in B cell growth, although the exact pathway is not clearly understood. It is likely that, apoptosis and differential control of cell cycle occurs with increased expression of *HNF1B* in pancreatic B cells, underlying its importance in B cell growth, although the exact pathway is still unclear. Protein tyrosine phosphatase‐BL (PTP‐BL or ptpn13), an HNF1B modulated protein in the B‐cell has been identified conjointly with its function in INS‐1 B cells.^59^ Welters and Morgan observed elevated PTP‐BL protein levels on subsequent induction of HNF1B expression in INS‐1 Flp‐In T‐Rex cell.^60^ Amplified HNF1B protein expression leads to compromised insulin secretion, augmented apoptosis and a decline in cell proliferation in INS‐1 cells ^60^ but the investigation of these responses showed their differential sensitivity to PTP‐BL. The results from this study indicated that Wnt signalling pathway modulating mature B‐cell growth is regulated by HNF1B (Figure 2).^59^

Osteopontin (OPN) gene expression has been found to be elevated in ovarian CCC and its expression to be associated with HNF1B overexpression.^61^ OPN comprises of *HNF1B* functional binding sites in its promoter stretch, validating as a direct target gene of *HNF1B*.^62^ OPN plays a crucial role in tumorigenicity by inhibiting apoptosis or activating matrix‐degrading proteases.^63^ Research on ovarian CCC has suggested *HNF1B* to be at the centre of a functional circuit, identifying susceptible targets of *HNF1B*.^64^ Chk1 (Checkpoint kinase 1) protein has been observed to be stimulated in the HNF‐1B‐overexpressing CCC cells. Chk1 has a role in the cellular existence pathways which augment DNA damage repair.^65^ The inhibition of Chk1 expression specifically chemosensitizes *HNF1B* ‐overexpressing CCC cells *in* *vitro*, underlying its importance as a novel therapeutic target in *HNF1B* positive cells.^64^ Senkel et al identified *HNF1B* regulated genes in the human embryonic kidney cell line (HEK293) and identified eight genes to be upregulated in ovarian CCC in comparison to other subtypes. Increased expression of *HNF1B* caused deregulation of genes including *SPP1*, *DPP4*, *SAH*, *RBPMS*, *CD24*, *NID2*, *LAMB1*, *RHOB* and *SOX9* in ovarian CCC.^62^ *SPP1* and Dipeptidyl Peptidase 4 (*DPP4*) had HNF1B binding sites in their promoter stretch, identifying them as a direct target. Additional studies in ovarian CCC revealed 22 genes as downstream targets of HNF1B.^66^ Furthermore, it has also been demonstrated that HNF1B could be associated with the genetic alterations most likely to be involved in the essential function of cell physiology of Ovarian CCC, such as oxidative stress (*DPPIV*, *ACE2*, Collectrin, *TFPi2*, *Octamer4*, *PAX8*), detoxifixation pathway, metabolism (GLUT2, ALDOB), adhesion and signal transduction (MAP3 K5/ASK1, mTOR). This genetic profile could lead to validation amongst stages of OCC in comparison to histological subtypes. It also highlights HNF1B as a unique molecular signature for pathophysiology of ovarian CCC. Additionally, Wiedmann and group characterised the interaction of DNA binding domain of HNF1B to Importin‐α isoforms using pull‐down assays. Also, HNF1B nuclear localisation signal (NLS) identified through biophysical techniques elucidated its role in facilitating the interactions of NLS peptide to Importin‐α.^67^ This has opened new avenues for developing new therapeutics against HNF1B selectively targeting the HNF1B‐Importinα interaction.

HNF1B has been observed to be directly regulating *HNF4α* in humans.^68^ Various pathways related to *HNF1B* need to be explored which could provide new insights to developing anticancer agents targeting these *HNF1B* regulated circuits. HNF1B induced expression of clotting factors in tumour cells, including elements involved in clotting cascade like prothrombin, fibrinogen, factor XIII, contributing to prothrombotic state in malignancy.^69^ The findings also indicated starch and sucrose metabolism genes as HNF1B targets. Xu et al identified a putative binding site in NNMT (Nicotinamide N‐methyltransferase) gene promoter region, which is greatly expressed in papillary thyroid cancers including the cell lines.^70^ These findings suggest HNF1B be a transcriptional activator of *NNMT* gene expression in some papillary thyroid cancers.

Co‐immunoprecipitation experiments by Choi et al indicated zyxin, a focal adhesion protein, as a novel interacting partner of *HNF1B* in renal epithelial cells.^71^ The study established the importance of an additional LIM domain of zyxin interacting with *HNF1B*. Additionally, it established the role of zyxin bound to CREB Binding Protein (CBP) stimulating the transcription of *HNF1B*. Co‐localization of zyxin with *HNF1B* was observed in the nucleus.^71^ Increased expression of zyxin leads to the transcriptional activity of *HNF1B*, on the contrary siRNA (RNA mediated) silencing of zyxin impeded the *HNF1B*‐dependent transcription. Expression of dominant‐negative mutant HNF1B, silencing of zyxin, resulted in reduced EGF‐induced cell migration. These results suggest a novel route for regulating *HNF1B* which is vital for renal epithelial differentiation.^72^ Previous studies have reported that *HNF1B* is essential for renal tubulogenesis by regulating the gene expression of *SOCS3*.^73^ This pathway showcases another route through which *HNF1B* might control tubulogenesis throughout kidney development. Also, the initial decline in *HNF1B* expression is linked with the over‐expression of one of its target genes, *SOCS3*, which is a requisite for renal repair. During developmental stages, HNF1B as a transcription factor has been identified to control a network of genes involved in duct morphogenesis.^74^ Inactivation of *HNF1B* by *Cre‐Sox9* recombination was found to cause chronic pancreatitis with dilation of ducts, acinar‐to‐ductal metaplasia and lipomatosis.^75^ Inactivated *HNF1B* in mouse ductal cells was seen to decrease the expression of *Prox1*, *Pkhd1*, *Spp1* and *Cys1*, which play an important role in maintaining tubule structure. This led to chronic pancreatitis and formation of neoplasia through *KRAS* activation in mature acinar cells. This study identifies HNF1B as a potential tumour suppressor gene in pancreatic cancer.

Hypoxia is regularly attributed to various tumours linked by disease progression along with treatment resistance.^76^ Hypoxia‐inducible factor 1 (HIF‐1) complex regulates many oxygen‐responsive genes. HIF‐1α overexpression, evident with accumulated immunostaining, has been described in various human cancers (including prostate cancer) during their metastases.^77^, ^78^ Current evidence proposes that HIF‐1 is engaged with notch‐responsive promoters in hypoxic conditions to initiate transcriptional targets.^79^ Hypoxia induces initial up‐regulation of *HNF1B* from 1 to 24 hours *in vitro*, independent of the HIF‐1α expression. When continued, hypoxia‐induced *HNF1B* down‐regulation while normoxia led to *HNF1B* normalization.^80^ Early reduction in *HNF1B* expression has been linked with transient overexpression of its target gene *Soc3*, which is vital for renal repair.^81^ Additionally, in a recent study by Li et al on polycystic kidney disease (PKD), *HNF1B* was shown to have been regulated by p53S, a mutant version of the tumour suppressor *p53*.^82^ RNA‐seq analysis to understand PKD in p53 mutant mice, revealed the suppression of *PKD1*, *PKD2*, *Pkhd1* (polyductin) and *HNF1B* expression. The data suggested developing a new gene signature for clinical diagnosis and prognosis of PKD highlighting the role of HNF1B in this disease. Igarashi et al demonstrated competitive DNA binding of HNF1B and B catenin in renal cells, affecting Wnt signalling in the kidney. Higher DNA occupancy by B catenin was observed in HNF1B mutant cells. This research showed that degradation of HNF1B in renal epithelial cells led to the deregulation of the Wnt signalling pathway.^83^

All these different studies have highlighted the targets and pathways involved with HNF1B, highlighting its role in different diseases. Although some of the studies have targeted the expression of HNF1B, none of them has provided insights into the role of transcript variants/isoforms. HNF1B expression seems to play an important role in *KRAS* activation for pancreatic tumour, highlighting the need for additional studies to develop therapeutic strategies improving survival in PDAC (pancreatic ductal adenocarcinoma) patients. Some of the common targets pan‐cancer like *p53*, *KRAS*, *Pkhd1*, *Chk1* and other targets/associated genes need to be studied in through large cohort patient sample studies to check their efficacy in developing novel strategies targeting *HNF1B*.

## 5 ROLE OF HNF1B IN CANCER

### 5.1 Tumour‐suppressive function

HNF1B is of the identified gene/s as per pathway analysis of GWAS data carried out by Li et al.^84^ HNF1B being a transcription factor, monitors development and differentiation in embryonic pancreas while also maintaining pancreatic homeostasis. PDX1, NR5A2, HNF1A and HNF1B function collectively carrying out regulated feedback circuit nursing pancreatic development and differentiation. There have been reports of somatic mutations of the *HNF1A* gene amongst various human cancers signifying its role as the tumour suppressor gene. HNF1A is a main regulator for plasma protein fucosylation and plasma levels of C‐ reactive protein.^84^ By mutating HNF1A by small interfering RNA in hepatocellular carcinoma cells increases overexpression of numerous genes translating cell cycle and angiogenesis regulators, growth factors receptors and components of translational machinery.^84^ HNF1B has been confirmed as a common cause of monogenic disorders such as developmental renal disease.^85^ Janky et al also reflected HNF1A/B as top enriched genes involved in the functioning of normal pancreatic tissue in comparison to regulatory network Pancreatic Ductal Adenocarcinoma (PDAC). HNF1B has shown to be implicated in balancing the beta‐cell transcription factor network and is essential for glucose sensing or glycolytic signalling in the pancreatic beta cell.^86^ HNF1B was shown to be down‐regulated *in vitro* in PDAC cells functioning via microRNA mechanism implying has‐miR‐24/23a. HNF1B deregulation has shown to be associated with epithelial to mesenchymal transition which might occur due to the destabilisation of the E‐cadherin and B‐catenin initiating altered cadherin/catenin nexus implicating the Wnt‐targeted genes in PDAC.^87^ Janky et al predicted HNF1A/B to be amongst the top transcription regulators in normal pancreatic tissue. Loss of protein expression in malignant ductal cells of the pancreas suggested tumour suppressive roles of HNF1A/B in pancreatic cancer.^86^ Moreover, it has been demonstrated that HNF1B, a paralogue of HNF1A, acts as both a cofactor of HNF1A or compensate the loss of HNF1A activity.^88^

HNF1B transcription factor is essential in the regulation of gene expression for organs such as pancreas, kidney, liver and gut. Renal diseases are homogenous phenotype which is usually associated with the mutation in transcription factor. Mostly these deformities have pancreatic atrophy and exocrine dysfunction. It has been observed that patients suffering from HNF1B mutations have varying renal functions from normal until dialysis‐dependent/transplanted.^89^ Buchner et al highlighted the role of HNF1B in metastatic renal cell carcinoma and demonstrated levels of *HNF1B* mRNA expression drastically declines in metastatic tumours whereas patients with higher *HNF1B* mRNA levels would have a better prognosis.^90^ Another case study carried out by Grunfeld et al. suggested a germline mutation of HNF1B (46delC) was associated with cystic kidney disease and chromophobe renal cell carcinoma, whereas a somatic deletion of HNF1B was reported for renal tumour.^91^ Chromophobe renal cell carcinoma was found to be associated with patients having biallelic inactivation of hepatocyte nuclear factor 1 beta. The study further confirms the role of HNF1B expression in renal neoplasms and its potential as a diagnostic marker for Chromphobe renal cell carcinoma.^92^ Nephrologists have also identified autosomal dominant mutation of HNF1B linked with polycystic kidney disease, diabetic nephropathy and cystic kidney disease (CKD) of unknown cause. Moreover, there has been widespread screening for individuals lacking HNF1B and HNF1A. In most of the disease states, genes such as PKHD1 (polycystic kidney and hepatic disease 1) and UMOD (Uromodulin), two genes regulated by HNF1B have been found to be inactivated. Rebouissou et al suggested the role of HNF1B as tumour suppressor gene in CRCC via the regulation of PKHD1.^93^ On the other hand another study carried out by Gad et al highlighted mutations in BHD and TP53 which is responsible for sporadic CRCC and found extremely rare events related to HNF1B mutation.^94^ Latest report by Bartu et al reflected the consequences of somatic exonic mutations of HNF1B along with its role in the pathogenesis of kidney tumours emphasizing HNF1B could act as oncogene in papillary renal cell carcinoma (reduced HNF1B was exhibited along elevated tumour grade with T stage) whereas it may behave as a tumour suppressor in CCRCC and CHRCC (no mutations was observed whilst promotor methylation was present).^95^

Many studies have pointed *HNF1B* locus with respect to SNP associations, although expression studies have conflicting data of these risk alleles in prostate cancer. Functional studies suggested the role of *HNF1B* as a pro‐differentiation factor that represses epithelial‐mesenchymal transition (EMT) in unmethylated normal tissues. Once methylated, the activity of *HNF1B* as a tumour suppressor is lost in course to the development of prostate cancer. Yamamoto et al. established that *HNF1B* immunoreactivity contrasted significantly between CCC and other pathological features in both the ovary and the endometrium, proposing *HNF1B* to be an eminent marker for differentiating CCCs from additional lesions together; the ovary and the endometrium.^96^ It was revealed by Kao and colleagues that the overexpression of *HNF1B* is specific for ovarian CCC amongst ovarian carcinomas.^97^ Ablation of *HNF1B* expression in ovarian CCC cells leads to a substantial proliferation, while increased expression of *HNF1B* in the serous Ovarian cancer (OC) cell line decreased cell growth.^98^ Decreased expression of *HNF1B* could impart drug resistance in OC and that *HNF1B* may contribute to drug resistance through regulating four pathways comprising p53 signalling, focal adhesion and ErbB signalling in addition to apoptosis (Refer to Table 2).^99^

**TABLE 2:** Role of HNF1B as Oncogene/Tumour suppressor gene in different cancers

Cancer	Oncogene/Tumour suppressor gene	Pathway affected	Reference
Pancreatic cancer	Tumour suppressor gene	Apoptosis, pancreatic development, hedgehog, *Helicobacter* *pylori* lacto/neolacto and Th1/Th2 immune response	85, 86
Renal Cell carcinoma	Tumour suppressor gene	Invasive alteration and dedifferentiation	90, 91, 92, 93, 94, 95
Ovarian cancer	Tumour suppressor gene	Epigenetic silencing	97, 98, 99
Prostate cancer	Oncogene/tumour suppressor gene	Epigenetic silencing	^32^
Endometrial cancer	Oncogene	Reduced promotor activity	^13^
Breast cancer	Oncogene	Epithelial‐mesenchymal transition (EMT)	^108^
Kidney cancer	Oncogene	Late tubular separation	^104^

### 5.2 Oncogenic roles

*HNF1B* deletion has been observed to cause condensation of pancreatic multipotent progenitor cells (MPCs) owing to reduced proliferation and increased apoptosis. De Vas et al detected that the Notch signalling pathway is dysregulated in *Hnf1b* mutant pancreas, displaying a reduction in Delta‐like Canonical Notch Ligand 1 (*Dll1)* expression and increased expression of Hairy and Enhancer of split related basic helix‐loop‐helix (*Hey)* repressors.^100^ HNF1B was found to be upregulated in ovarian cell carcinomas and clear cell variant of ductal adenocarcinoma.^101^

Trisomy and tetrasomy of chromosome 17 have been highlighted as a cause for papillary renal cell tumours (RCT).^102^, ^103^ A comprehensive detailed analysis has shown *HNF1B* gene loci amplification in RCT; the protein being detected in papillary RCTs, mucinous tubular and spindle cell carcinomas (MTSCC) and metanephric adenomas.^104^ Differentiating tubules of the fetal kidney produced HNF1B protein, while high expression was observed in adult carcinomas of embryonal origin. The copy number changes and increased expression of the *HNF1B* gene were linked with late tubular separation in precursor lesions (Refer to Table 2).

*HNF1B* is associated with cancer cell proliferation, tumour progression, and castration‐resistant prostate cancer.^32^, ^105^ Painter et al. highlighted the role of the *HNF1B* protective rs11263763 SNP allele in EC with reduced promoter activity, indicating *HNF1B’s* oncogenic role.^13^ Similarly, Larson and colleagues performed trans‐EQTL studies and identified risk alleles increasing *HNF1B* expression, suggesting a probable oncogenic role in PCa.^106^ This study also found intronic rs3110641 SNP to be associated with changes in HNF1B isoform expression. Additional studies in breast cancer have also emphasized *HNF1B* overexpression in inducing EMT in epithelial NMuMG cells.^107^ A study corroborating key role of EMT in metastasis and therapeutic resistance has shed light on the prospect of developing new strategies targeting EMT signalling.^108^ However, contrasting evidence shows that inhibition of EMT does not prevent metastasis.^109^ Targeting transcription factors has always been a challenge with them being called ‘undruggable’. As previously discussed, lower levels of HNF1B in prostate cancer has now been associated with higher EZH2, promoting EMT and metastasis. A new therapeutic approach targeting this axis could help in treating tumour growth.^58^

## 6 HNF1B AS A BIOMARKER IN CANCER

*HNF1B* has been well characterised in liver, pancreas and kidney, while diagnosing its related disease has been challenging due to phenotypic variability.^110^ Among the first to identify the potential of *HNF1B* in hepatocellular carcinoma (HCC), Ninomiya et al highlighted the ratio of *HNF1A*/*HNF1B* expression in HCC tissues to be higher in well‐differentiated cases compared to undifferentiated and poorly differentiated cases.^111^ Similarly, Wang et al reported the ratio of *HNF1A*: *HNF1B* expression to be related to histologically differentiated disease.^112^ The expression of HNF1B was found to be higher in differentiated HCC compared to non‐cancerous tissues. These studies emphasize the potential role of *HNF1B* as a biomarker. Several studies have highlighted the role of HNF1 family members in regulating alpha‐fetoprotein (AFP) promoter expression during hepatic development and carcinogenesis.^113^, ^114^ Immunohistochemistry studies in liver transplant patients with HCC revealed that *HNF1B* has been associated with serum AFP level and *AFP* expression. Transcriptional regulation of *AFP* through *HNF1B* may function during different stages of HCC progression following recurrence. The expression of *HNF1B* in tumour tissue, thus, can predict relapse and mortality after transplantation.^115^ Shim et al. also monitored the relevance of HNF1B in hepatocellular carcinoma following liver transplantation which corresponded with the findings. Additionally, Yu et al. investigated the expression of *HNF1B* with clinicopathological features and prognosis in HCC and cholangiocarcinoma (ICC) patients.^116^ HNF1B expression was found to be positively correlated with recurrence in HCC indicating poor prognosis. However, no correlation was found with its expression in ICC and survival. Further studies need to be conducted on developing HNF1B as a prognostic marker predicting recurrence in HCC.

In the latest study by Nie et al., HNF1B expression was observed to be higher in uterine corpus endometrial carcinoma, bladder urothelial carcinoma and liver hepatocellular carcinoma and minimal expression levels in liver hepatocellular carcinoma, colon carcinoma, glioblastoma multiforme, kidney chromophobe and kidney renal clear cell carcinoma. HNF1B expression is associated along with heterogenous immune cell infiltration levels in distinct cancers.^117^ For instance, HNF1B expression is correlated with the infiltration grades of B cells, CD8+ T cells, CD4+ T cells, macrophages, neutrophils along with Dendritic cells in cancer. Patients were categorized into low and high HNF1B expression groups based on the CD4+ T cell, CD8+ T cell along with B‐cell levels, which were further grouped based on overall survival of cancer patients into different subsets. This study emphasizes the correlation amongst HNF1B and immune cells and the need for further investigation into the HNF1B expression for patients undergoing immunotherapy. Moreover, the group also demonstrated HNF1B mRNA dysregulation in different cancers that lead to differential expression of HNF1B leading to distinct prognosis.^117^

Pancreatic abnormalities have been tested for *HNF1B* mutations and its prospectus as a biomarker being widely debated in renal hyperplasia and cysts.^118^ Yang and colleagues investigated the role of HNF1B in PDAC as a diagnostic marker in a large cohort of 127 primary and 17 metastatic PDACs, 47 biliary adenocarcinoma and 231 pancreaticobiliary carcinomas.^119^ Majority of pancreatic and biliary epithelium carcinomas had an expression of *HNF1B*, with statistical analysis showing 84% sensitivity and 85% predictive value overall. The expression of *HNF1B* was associated with tumour size and grade highlighting the potential of the gene as a biomarker in PDAC. Likewise, *HNF1B* was shown to be part of five gene expression signature predictive of relapse in prostate cancer patients. Kaplan‐Meier analysis revealed that relapse of predictor markers are highly useful in the classification of patients into subgroups with distinct relapse‐free survival after therapy.^120^ Furthermore, Harries et al reported a probable role of alternatively spliced mRNA of *HNF1B* and *MSMB* genes in the cause of prostate cancer.^121^

Ovarian CCC has a poor prognosis of all the epithelial ovarian cancers.^122^ Immunohistochemical analysis showed HNF1B nuclear staining in clear cell carcinoma specimens but minimal nuclear staining in non‐clear cell carcinoma specimens. HNF1B may be an excellent CCC‐specific molecular marker.^123^ Apoptotic cell death was seen in TOV‐21G and JHOC‐5 ovarian CCC cell lines induced by reduction of *HNF1B* expression by RNA interference indicating that, *HNF1B* is an excellent CCC‐specific molecular marker and can also be used as the molecular target therapy for Ovarian CCC.^123^ Similarly, Huang and colleagues also showed expression of *HNF1B* in ovarian CCC patients with a statistical specificity of 76.5% and selectivity of 85.2%.^124^ In a study investigating *HNF1B* expression in ovarian clear cell tumours, there was a clear distinction of the levels of the protein being present between clear cell carcinomas vs non‐clear cell carcinoma, indicating its potential as a molecular marker for ovarian CCC irrespective of benign or malignant lesions.^61^ Cuff et al defined *HNF1B* as an extensive marker for clear cell phenotypes, endorsing a mechanistic association to glycogen aggregation and thrombosis. This outcome suggests a novel mechanism of tumour linked thrombosis centred on cancer cells directly producing clotting factors.^69^

*HNF1B* and ER (oestrogen receptor) may be used as a diagnostic panel to discriminate endometrioid from clear cell carcinoma besides serous carcinoma of the endometrium.^125^ CCC of the urinary tract has been identified as a rare malignancy, which mimics the clear cell carcinoma of the female genital tract morphologically.^126^ One of the studies described *HNF1B* as a biomarker in distinguishing clear cell adenocarcinomas of the bladder/urethra besides other primary tumours of the urinary bladder, specifically aggressive urothelial tumour with clear cell change.^127^ Davidson et al advocated the central part of *HNF1B* marker in serous effusion diagnosis, with the data suggesting HNF1B to be an important biomarker which may differentiate CCC from serous ovarian carcinoma and cells of mesothelial origin.^128^

In a nutshell, a wide array of expression changes in different cancers and its subtypes does point towards the role of this gene as a potential biomarker. However, even after identifying its involvement in development and progression to tumour relapse, the details about the regulatory pathways still are missing, further studies which highlight these missing links can help in finally helping the potential of HNF1B to become a biomarker in different cancers.

## 7 CONCLUSION

*HNF1B* was first reported as a potential candidate gene for MODY although extensive research indicated it as an important gene having a role in tumorigenesis. *HNF1B* profiles in different tumours throw light on different mechanisms governing *HNF1B* function and expression. GWAS identifying *HNF1B* risk loci and epigenetic alterations has unravelled the risk alleles and the role of DNA methylation and their potential as biomarkers for disease prognosis. The function of *HNF1B* as a potential tumour suppressor gene has highlighted its possible role as a therapeutic target followed by identifying the role of HNF1B as a unique marker for characterizing CCCs from other lesions in ovary and endometrium. Moreover, the expression of HNF1B has been associated with immune cell infiltration which in turn influences the prognosis of immune cells in some cancers highlighting HNF1B as a potential immunotherapeutic target. Likewise, evaluating the functional role of the transcript variants may be crucial for developing accurate biomarkers and effective therapeutic strategies. Regulatory pathways and mechanisms involving *HNF1B* still need to be elucidated and studied in depth to explore the networking of the HNF family. Understanding splice variants of *HNF1B* and their role will broaden the scope of pursuing new downstream targets, its associated signalling pathways and transcriptional efficacy governing different gene sets. In‐depth characterization of *HNF1B* and its splice variants are warranted to compliment its ever‐growing importance in different cancers.

## CONFLICT OF INTEREST

The authors declare that the research was conducted in the absence of any commercial or financial relationships that could be construed as a potential conflict of interest.

## AUTHOR CONTRIBUTIONS

All authors listed have made a substantial, direct and intellectual contribution to the work, and approved it for publication.

## Funding information

This project is funded by Cancer Australia PdCCRS Young Investigator grant to JB. JB was supported by NHMRC Career Development Fellowship, SS by Advance Qld Fellowship and a John‐Mills Young Investigator Award, SC by QUTRPA scholarship.
